# Video Versus Direct Laryngoscopy in Novice Intubators: A Systematic Review and Meta-Analysis

**DOI:** 10.7759/cureus.29578

**Published:** 2022-09-25

**Authors:** Shreya Nalubola, Evan Jin, Elizabeth D Drugge, Garret Weber, Apolonia E Abramowicz

**Affiliations:** 1 Anesthesiology, New York Medical College, Valhalla, USA; 2 Anesthesiology, Westchester Medical Center, Valhalla, USA; 3 Public Health, New York Medical College School of Health Sciences and Practice, Valhalla, USA

**Keywords:** video laryngoscopy, intubation, novices, airway management, direct laryngoscopy

## Abstract

Video laryngoscopy (VL) is increasingly used in airway management and has been shown to decrease the rate of failed intubation in certain clinical scenarios, such as difficult airways. Training novices in intubation techniques requires them to practice on living patients; however, this is less than ideal from a safety perspective given the increased risk of complications after multiple attempts or failed intubation by inexperienced trainees. One setting in which VL may be beneficial is in training, although whether these devices should be used among novices instead of direct laryngoscopy (DL) remains unclear. The purpose of this systematic review and meta-analysis is to compare the outcomes of VL and DL when used by novices to perform intubation in the operating room. The secondary aims are to correlate outcomes with different types of VLs and with different types of novices, such as medical students, residents, and non-anesthesiology trainees.

Databases were searched for studies that compared the outcomes of VL versus DL in endotracheal intubation performed by novices on patients with expected normal airways and no history of difficult intubation or cervical spine instability undergoing general anesthesia in the operating room. The primary outcome was the initial success rate. The secondary outcomes were time to intubate and the number of unintended esophageal intubations. A meta-analysis was performed to determine the difference, if any, in outcomes between VL and DL. Sub-analyses were also performed after the stratification of data by the type of VL used and the type of novice.

Ten studies were included with 1,730 intubations. Studies varied by VL type and novice type. The overall results from the meta-analysis demonstrated an increased success rate and decreased time to intubate with VL compared to DL. Four studies showed a reduction in esophageal intubation with VL compared to DL. Sub-analysis by VL type showed that improved outcomes with VL over DL were maintained only with the use of channeled VLs rather than non-channeled VLs. Sub-analysis by novice type showed that improved success rates with VL over DL were maintained only among medical students.

Novices may have a higher initial success rate and faster intubation time when using a channeled VL compared to DL. Medical students also show improved success rates when using VL rather than DL, while residents and other types of novices do not. These findings may help guide clinicians in determining the most effective devices to use when teaching airway management while also maintaining the highest possible level of patient safety.

## Introduction and background

Over the past 20 years, video laryngoscopy (VL) has emerged as an important tool in airway management, rivaling the use of traditional direct laryngoscopy (DL) in oral endotracheal intubation [1]. VLs may reduce the rate of failed intubations; thus, there has been a shift toward using these devices in certain clinical scenarios, such as difficult airways or cases in which a rescue device is indicated, in order to provide the most effective patient care [2,3]. In particular, VL is a useful tool in intubation training by incorporating an external image viewing screen that provides a superior glottic view and allows instructors to share feedback in real time. Teaching proper intubation techniques with these devices may therefore lead to better outcomes and decrease the risk of complications in patients [4]. While novices such as medical students and residents are often supervised when intubating, the potential for patient harm remains due to the high-risk nature of this procedure, and VL may be helpful in finding the crucial balance between patient care and teaching technical skills in clinical practice [5]. VL is also suspected to decrease cognitive overload, which novices are particularly susceptible to as they start to perform challenging procedures such as intubation [6].

When comparing VL and DL, it is important to note that many different types of VLs exist. Earlier VL models such as Glidescope favor the non-channeled blade, in which the VL is held in one hand, while the endotracheal tube is maneuvered with the other. The development of VLs such as Airtraq with an integrated channel, or space into which the endotracheal tube is inserted and made visible on camera, is argued to further decrease cognitive load for those who have not yet gained familiarity with maneuvering laryngoscopes [7]. In addition, different novices such as medical students, anesthesiology residents, or other non-anesthesia trainees often have variations in baseline skill levels that should be considered. Quantifying the outcomes of VL versus DL in novices at various educational levels can shed light on when to use one laryngoscope over another in stages of training.

This study is a systematic review and meta-analysis of the use of VL versus DL in novices with minimal experience in intubation. The primary aim is to determine whether novices perform better when using VL over DL as measured by the initial success rate and time to intubate. The secondary aims are to assess the performance of novices using different types of VLs versus DL and to determine if various types of novices have distinct outcomes when intubating with VL versus DL. Determining whether novices have increased initial proficiency with one type of tool has implications for improving patient safety by decreasing the risk of complications for the patient [8].

## Review

Methods

The study was performed according to the Preferred Reporting Items for Systematic Reviews and Meta-Analyses (PRISMA) statement [9]. The review protocol was registered with the International Prospective Register of Systematic Reviews (PROSPERO) (CRD42021293173).

Search Strategy

A literature search was performed using MEDLINE/PubMed, EMBASE, Web of Science, and Cochrane Library for studies assessing VL and DL performance among novice intubators in the operating room. The search occurred from October 2021 to January 2022. References of all selected articles were also searched to ensure that relevant studies not found in the initial search of the databases were included. The full electronic search strategies can be found in the Appendix.

Two authors reviewed texts independently and screened records by title, abstract, and keywords for potential eligibility (SN and EJ). Data was collected using Microsoft Excel 2020 (Microsoft Corp., Redmond, WA, USA). Full-text articles were reviewed in detail to ensure all inclusion criteria were met and assessed for risk of bias and quality of evidence. Disagreements were resolved by a third author (GW).

Selection Criteria

Inclusion criteria included studies of adult patients only (>18 years of age) with intubators who were considered novices or not well-trained in VL and/or DL, normal airways intubated in the operating room, and confirmed use of a video screen along with the VL. Exclusion criteria included papers that were not available in English, did not pertain to learning or training, did not have a success rate as an outcome, did not include a comparison of VL to DL, were literature reviews, case reports, abstracts, editorials, or comments, or did not have full-text available. There was no restriction placed on the geographic location or time at which the study was conducted.

Risk of Bias

Two authors (SN and EJ) independently reviewed each selected study for risk of bias using the Cochrane Collaboration’s tool for assessing risk of bias [10]. The AUB-KQ1 was used to assess the bias in the included randomized controlled trials (RCTs). The ROBINS-I tool was used for non-RCT studies.

Data Extraction

An electronic data collection form (Microsoft Excel 2020) was used to collect raw data from each study. Data were extracted from each paper by two authors (SN and EJ) working independently who then cross-checked the data independently as well. Finally, any discrepancies were resolved by a third author (GW).

Outcomes

Primary and secondary outcomes were decided a priori. Individual study data for all outcomes were extracted. The success rate of intubation, particularly on the first pass or the fewest number of recorded attempts, was the primary outcome used to compare VL and DL. Studies have shown that the risk of complications increases with the number of subsequent intubation attempts [8,11]. First-pass success rate therefore may serve as a surrogate measure for the risk of adverse events [12,13]. This outcome measure was separated in subgroup analyses by the different types of novices included in the studies and by VL type.

The secondary outcomes were total time to successful intubation and incidence of esophageal intubations. Inability to intubate the patient and subsequent prolonged apnea can lead to adverse outcomes such as hypoxia and increase patient mortality [8]. Time to secure the airway was also used in subgroup analyses by novice type and VL type. No subgroup analyses were performed on the incidence of esophageal intubations due to the limited number of studies that reported complete data on this outcome (four).

Statistical Analysis

Meta-analyses were performed to assess pooled outcomes for mean differences for continuous outcomes (time to intubate in seconds) and log risk ratios (RR) with a 95% confidence interval (CI) for ordinal outcomes (success rate and the number of esophageal intubations). A random-effects model with restricted maximum likelihood (REML) estimation was used to analyze the data with Stata 17.0 (StataCorp LLC, College Station, Texas, USA). Additionally, the I² method with a pooled prevalence estimate was used to calculate statistical heterogeneity. A value above 80% was considered significantly heterogeneous, while a value above 50% was considered moderately heterogeneous. P values were considered statistically significant if less than 0.05.

Results

**Figure 1:**
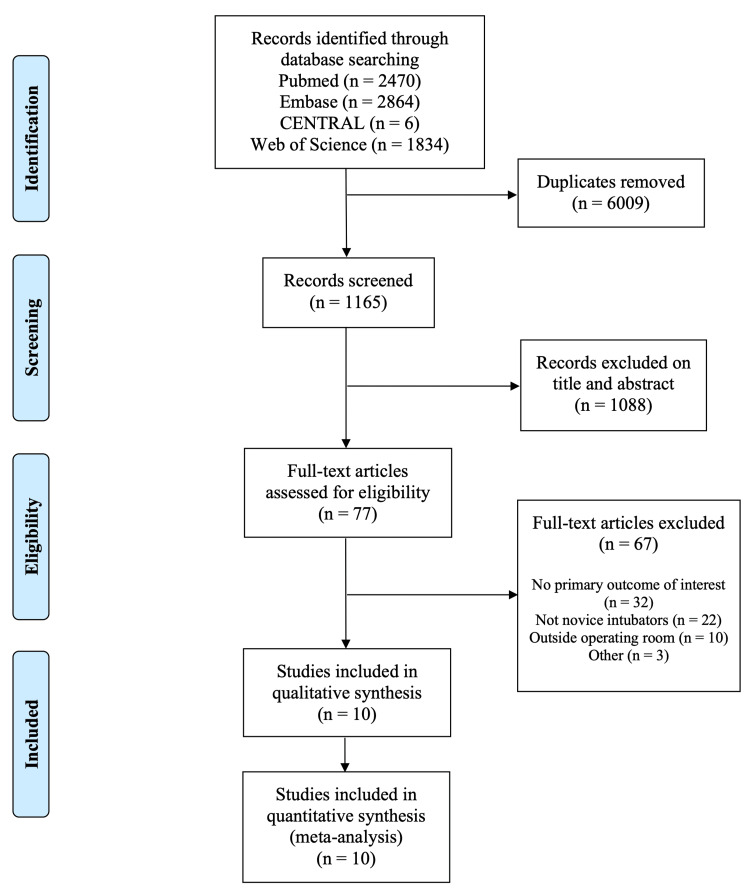
Preferred Reporting Items for Systematic Reviews and Meta-Analyses (PRISMA) flow diagram. The PRISMA flow diagram demonstrates the study selection process and the reasons for the exclusion of identified records.

The process for study selection is demonstrated in the PRISMA flow diagram (Figure 1). A total of 10 studies met the inclusion criteria [14-23]. One article was excluded for consistency due to its comparison to DL using the straight Miller blade instead of the curved Macintosh blade [24]. All 10 studies were included in the meta-analyses of success rate and time to intubate. Nine studies reported first-pass success rate, while one paper reported the overall success rate of two attempts [14]. Four of the 10 studies reported the incidence of esophageal intubation [16,17,21,22].

Study Characteristics

Study characteristics are shown in Table 1. Intubator populations were varied and consisted of medical students, first-year residents, first-year anesthetists, non-anesthesiology physicians, non-anesthesiology residents, paramedics, first-year house staff, and nurses. The types of novices in the selected studies were divided into medical students, first-year residents, and other novice clinicians in the sub-analyses. All studies required some form of basic training in intubation prior to participation.

The types of VLs used in the selected studies were divided into non-channeled (Glidescope, McGrath, and TruView) and channeled (Airtraq and Pentax) types in the sub-analysis. All studies compared only one type of VL, either channeled or non-channeled, to DL.

The primary and secondary outcomes measured are also shown in Table 1. Five studies defined time to successful intubation as the insertion of the blade to the appearance of the first upward wave on the capnograph [14,18,19,21,22]. Two studies defined time to successful intubation as the opening of the mouth to the first normal wave [20,23]. Hirabayashi and Seo defined the time in seconds from the interruption of intermittent positive pressure ventilation to the connection of the endotracheal tube to an anesthesia circuit [16,17]. Lastly, Di Marco et al. defined the time from the insertion of the blade to the visualization of the vocal cords [15].

**Table 1:** Study characteristics. Studies are listed alphabetically. RCT: randomized controlled trial; TTI: time to intubate; VL: video laryngoscopy; DL: direct laryngoscopy; SD: standard deviation ^a^Bakshi et al. [14] had an experimental group within their larger study that consisted of complete novices to intubation; this group (“novices to intubation” or “NTI”) was isolated and included in our meta-analysis. ^b^Nouruzi-Sedeh et al. [20] included a mixed group of novices that was analyzed with the non-medical student, non-resident intubators in the subgroup analysis. ^c^Study design refers to whether there was a crossover design with intra-subject participation in VL and DL or separation of intubators into groups performing solely VL or solely DL.

Author	Year	Study type	Country	Total number of intubations	Intubator type (number)	Study design^c^	Prior training with intubation	VL type (blade type)	Total VL intubations	VL success (%)	Total DL intubations	DL success (%)	VL TTI (SD)	DL TTI (SD)	Esophageal intubation VL	Esophageal intubation DL
Bakshi et al. [14]^a^	2015	RCT	India	42	NTI group (residents with no experience) (6)	Crossover	Manikin training in DL and VL	McGrath, TruView (non-channeled)	28 (14 McGrath, 14 TruView)	21 (8 McGrath, 13 TruView) (75)	14	12 (85.7)	115 (5)	103 (5)	Not recorded	Not recorded
Di Marco et al. [15]	2011	RCT	Italy	108	First-year residents (18)	Separate	Manikin training in DL and VL	Airtraq (channeled)	54	47 (87)	54	43 (79.6)	40 (23)	59 (26)	Not recorded	Not recorded
Hirabayashi et al. [16]	2009	Prospective cohort	Japan	520	Non-anesthesiology residents (48)	Separate	Two-month anesthesia training course, manikin training in DL and VL with minimal clinical use	Pentax (channeled)	264	253 (95.8)	256	179 (69.9)	44 (19)	71 (44)	0	18
Hirabayashi et al. [17]	2009	RCT	Japan	200	Non-anesthesiology novice physicians (43)	Separate	Variable amount of anesthesia training involving minimal clinical use of DL, manikin training with VL and DL	Airtraq (channeled)	100	95 (95)	100	79 (79)	51 (17)	67 (43)	0	10
Kim et al. [18]	2018	RCT	South Korea	220	First-year residents (11)	Crossover	Manikin training in DL and VL	Pentax (channeled)	110	104 (94.5)	110	89 (80.9)	33 (8)	44.7 (5.6)	Not recorded	Not recorded
Liu et al. [19]	2016	RCT	China	177	First-year trainee anesthetists (9)	Crossover	10-30 intubations on actual patients with DL, manikin training with VL	McGrath (non-channeled)	88	80 (90.1)	89	84 (94.4)	30.6 (14.8)	28.7 (12.3)	Not recorded	Not recorded
Nouruzi-Sedeh et al. [20]^b^	2009	Comparative clinical study	Germany	160	Inexperienced trainees: 8 paramedics, 4 first-year house staff, 4 nurses, 4 medical students (20)	Crossover	Manikin training in DL and VL	Glidescope (non-channeled)	100	93 (93)	100	51 (51)	63 (30)	89 (35)	Not recorded	Not recorded
Park et al. [21]	2010	RCT	South Korea	74	Medical students (37)	Crossover	Manikin training in DL and VL	Airtraq (channeled)	37	32 (86.5)	37	19 (51.4)	58.1 (23.1)	90.3 (39.8)	2	7
Peirovifar et al. [22]	2014	RCT	Iran	40	Medical students (40)	Separate	Manikin training in DL and VL	Glidescope (non-channeled)	20	16 (80)	20	12 (60)	31.5 (3.59)	37.55 (3.48)	0	2
Zhao et al. [23]	2014	RCT	China	149	Medical students (26)	Crossover	Manikin training in DL and VL	Airtraq (channeled)	74	65 (87.8)	75	50 (66.7)	68 (21)	96 (22)	Not recorded	Not recorded

Results from the risk of bias assessment are shown in Table 2. The risk of bias in the randomized controlled trials (RCTs) was assessed as low or unclear across all domains except the blinding of participants and personnel. The risk of bias of the non-RCTs was assessed as low or moderate across all domains.

**Table 2:** Risk of bias assessment. The AUB-KQ1 was used to assess the bias in the included RCTs. The ROBINS-I tool was used for non-RCT studies. All studies scored the same with respect to each category. RCTs: randomized controlled trials

RCT criteria	Selection bias (random sequence generation)	Selection bias (allocation concealment)	Reporting bias (selective reporting)	Performance bias (blinding of participants and personnel)	Detection bias (blinding of outcome and assessment)	Attrition bias (incomplete outcome data)	Other sources of bias
Results from all RCT studies [14, 15,17-19,21-23]	Low	Low	Unclear	High	High	Low	None
Non-RCT criteria	Bias due to confounding	Bias in the selection of participants into the study	Bias in the classification of interventions	Bias due to deviations from intended interventions	Bias due to missing data	Bias in measurement outcomes	Bias in the selection of the reported result
Results from all non-RCT studies [16,20]	Low	Low	Low	Low	Low	Moderate	Low

Success Rate

**Figure 2:**
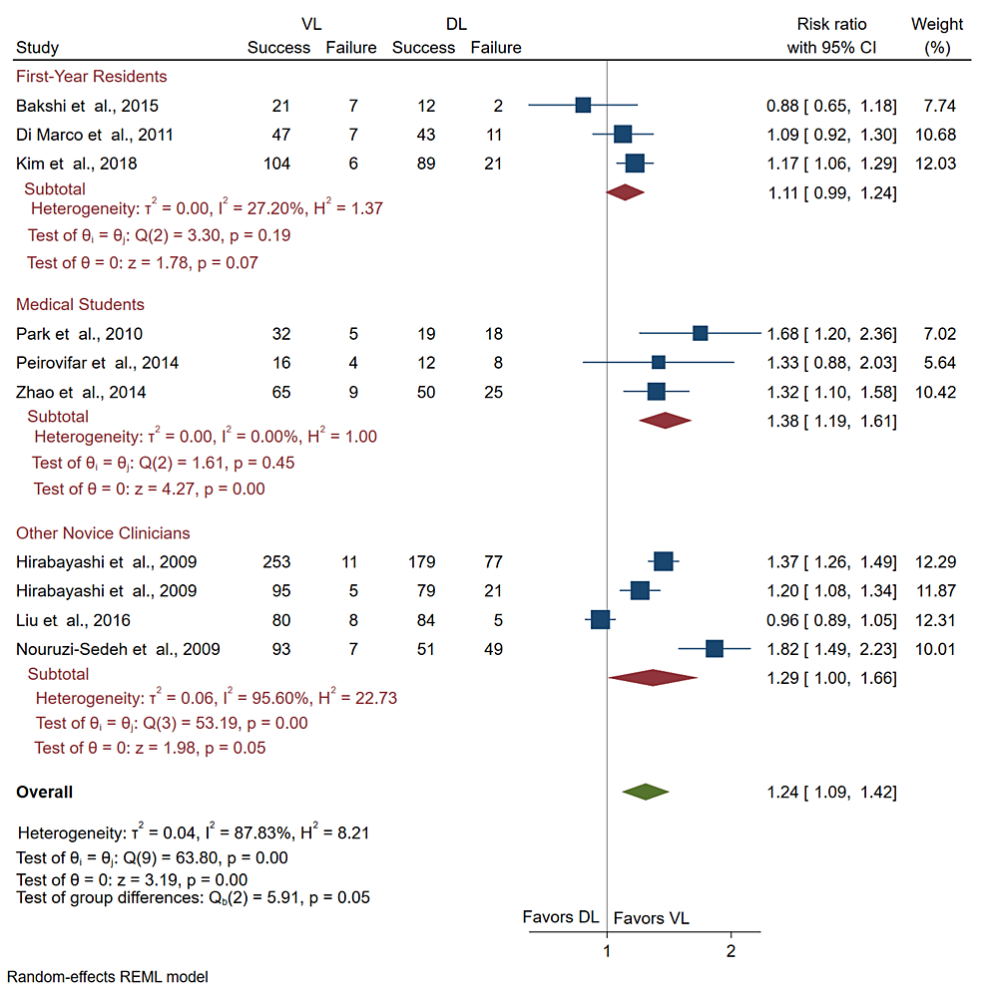
Forest plot of pooled risk ratios for the success of novices using VL or DL. The figure demonstrates the success rates of VL versus DL when stratified by the type of novice. CI: confidence interval; VL: video laryngoscopy; DL: direct laryngoscopy Bakshi et al. [14], Di Marco et al. [15], Hirabayashi et al. [16,17], Kim et al. [18], Liu et al. [19], Nouruzi-Sedeh et al. [20], Park et al. [21], Peirovifar et al. [22], Zhao et al. [23]

Ten studies with 1,730 intubations (875 VL and 855 DL) were included in the comparison of success rates. There was a significant increase in the initial success rate when VL was used rather than DL (RR = 1.24; 95% CI = 1.09, 1.42) across all studies (Figure 2).

Comparison of success rate by novice subgroup: After subgroup analysis by novice type, the initial success rate for medical students was higher for those using VL over DL (RR = 1.38; 95% CI = 1.19, 1.61). The differences in the initial success rate were not statistically significant for the other novice clinician subgroup (RR = 1.29; 95% CI = 1.00, 1.66) or the first-year resident subgroup (RR = 1.11; 95% CI = 0.99, 1.24) (Figure 2).

**Figure 3:**
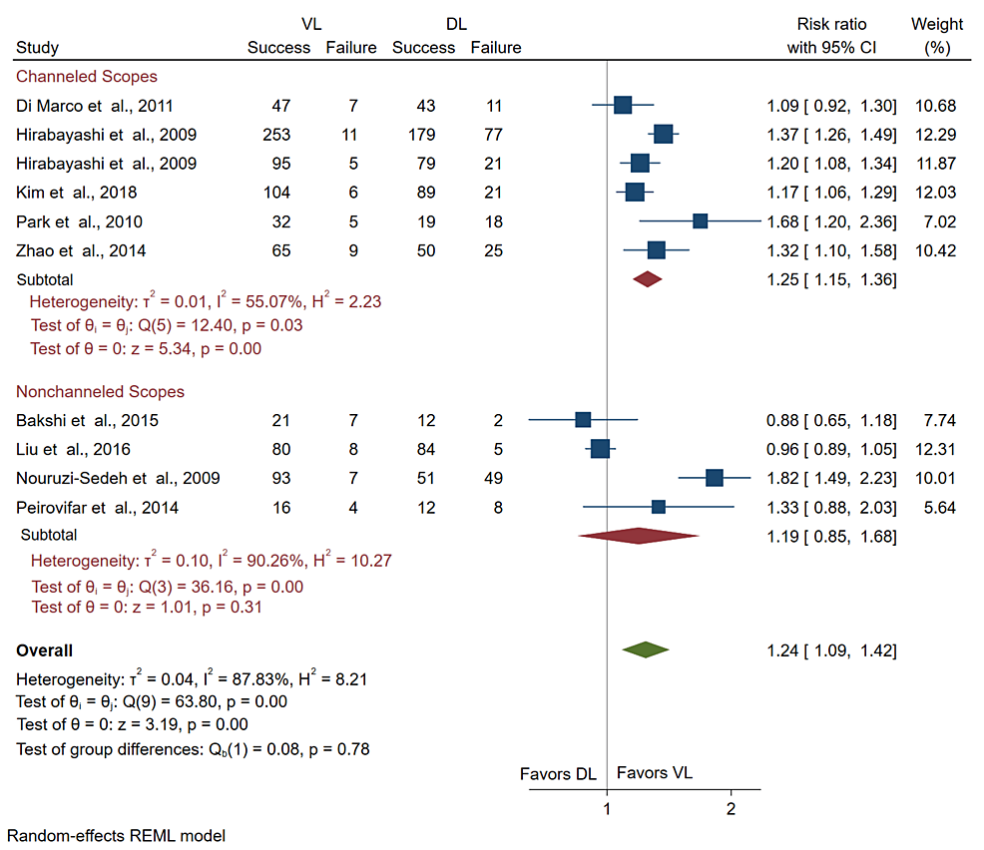
Forest plot of pooled risk ratios for the success of novices using channeled VL or non-channeled VL versus DL. The figure demonstrates the success rates of VL versus DL when stratified by the type of VL. CI: confidence interval; VL: video laryngoscopy; DL: direct laryngoscopy Bakshi et al. [14], Di Marco et al. [15], Hirabayashi et al. [16,17], Kim et al. [18], Liu et al. [19], Nouruzi-Sedeh et al. [20], Park et al. [21], Peirovifar et al. [22], Zhao et al. [23]

Comparison of success rate by VL subgroup: Subgroup analysis of channeled and non-channeled VL usage resulted in a significant increase in success rate with the use of the channeled scopes compared to DL (RR = 1.25; 95% CI = 1.15, 1.36). In contrast, the results were not significant when comparing the non-channeled VLs to DL (RR = 1.19; 95% CI = 0.85, 1.68) (Figure 3). We interpret these results to suggest that when stratified by channeled and non-channeled VLs, only channeled VL usage significantly increases success rate compared to DL.

Time to Intubate

**Figure 4:**
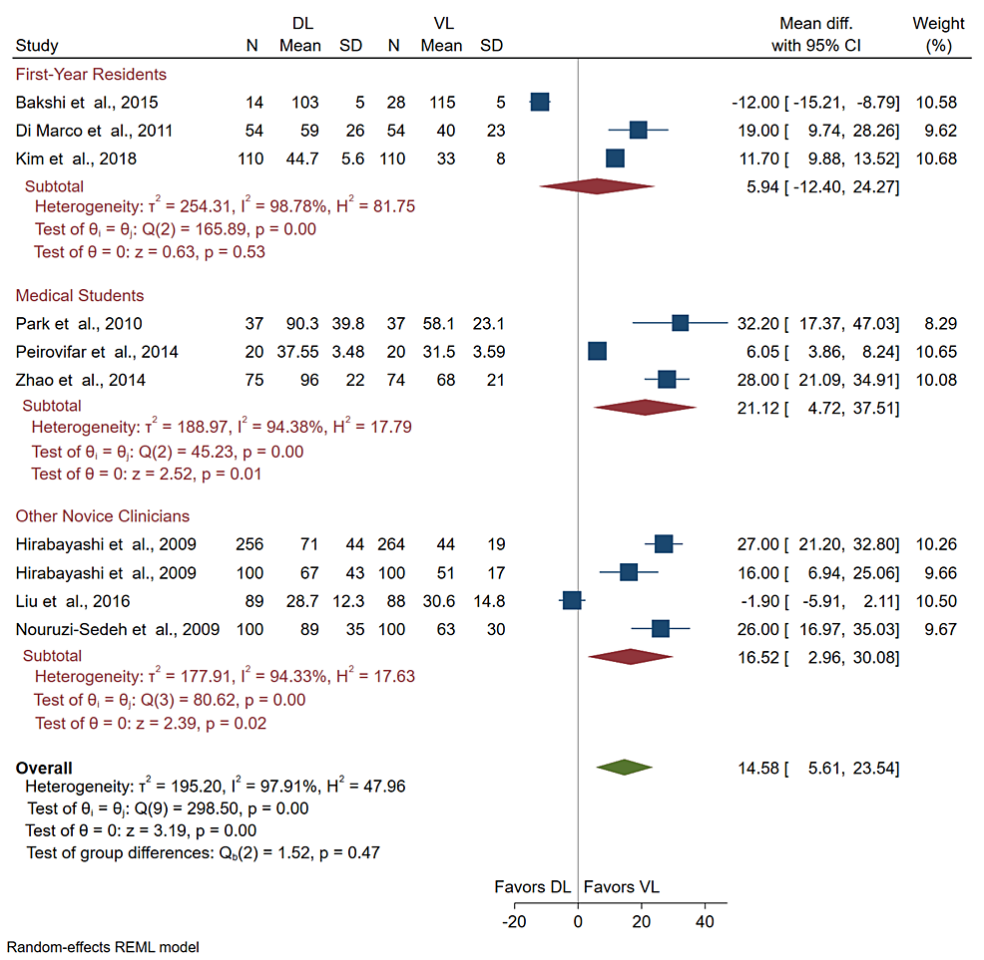
Forest plot of the difference in the mean time to intubate (seconds) for novices using VL or DL. CI: confidence interval; VL: video laryngoscopy; DL: direct laryngoscopy Bakshi et al. [14], Di Marco et al. [15], Hirabayashi et al. [16,17], Kim et al. [18], Liu et al. [19], Nouruzi-Sedeh et al. [20], Park et al. [21], Peirovifar et al. [22], Zhao et al. [23]

Ten studies with 1,730 intubations (875 VL and 855 DL) were included in the comparison of time to intubate. The mean time to intubate in seconds was significantly higher with DL compared to VL in the overall analysis (mean difference (DL - VL) = 14.58 seconds; 95% CI = 5.61, 23.54) (Figure 4).

Comparison of time to intubate by novice subtype: The time to intubate was significantly greater when using DL rather than VL for medical students (mean difference (DL - VL) = 21.12 seconds; 95% CI = 4.72, 37.51) and for other novice clinicians (mean difference (DL - VL) = 16.52 seconds; 95% CI = 2.96, 30.08). There was no significant difference in time to intubate among residents (Figure 4).

**Figure 5:**
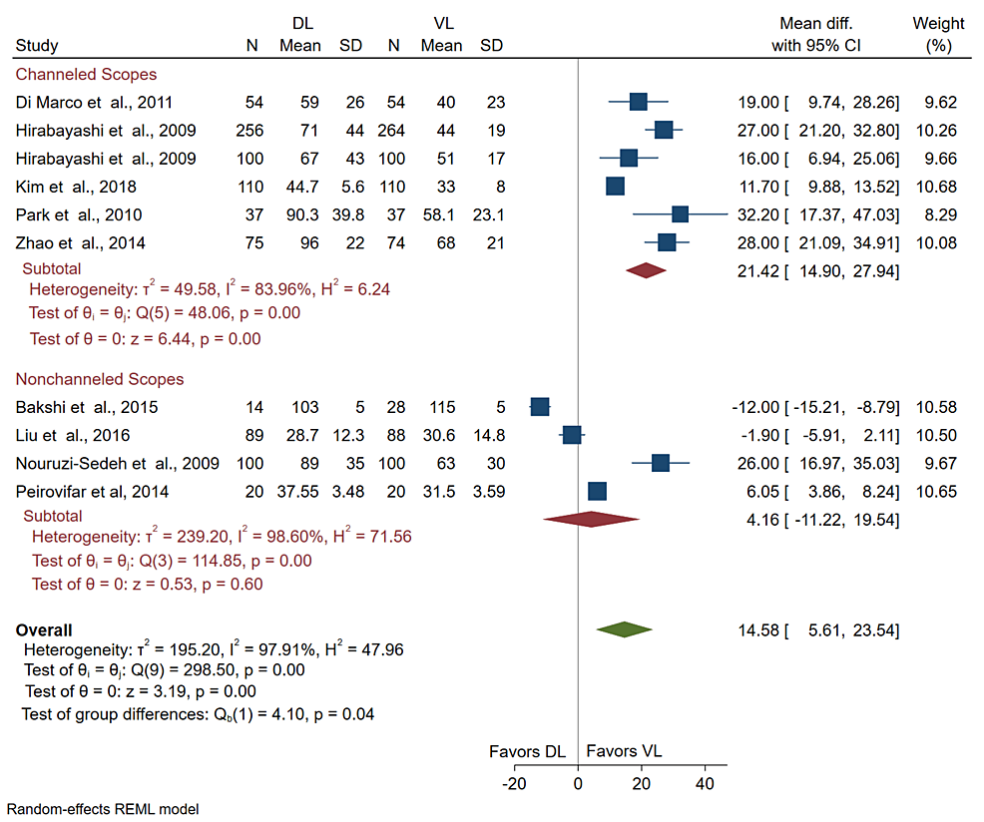
Forest plot of the difference in the mean time to intubate (seconds) for novices using channeled VL or non-channeled VL versus DL. CI: confidence interval; VL: video laryngoscopy; DL: direct laryngoscopy Bakshi et al. [14], Di Marco et al. [15], Hirabayashi et al. [16,17], Kim et al. [18], Liu et al. [19], Nouruzi-Sedeh et al. [20], Park et al. [21], Peirovifar et al. [22], Zhao et al. [23]

Comparison of time to intubate by VL subgroup: Results comparing channeled and non-channeled VLs versus DL demonstrated a significant increase in time to intubate with the use of DL over the channeled VLs (mean difference (DL - VL) = 21.42 seconds; 95% CI = 14.90, 27.94). Conversely, there was no significant difference in time to intubate with the use of the non-channeled VLs (mean difference (DL - VL) = 4.16 seconds; 95% CI = -11.22, 19.54) (Figure 5). We interpret these results to suggest that when stratified by channeled and non-channeled VLs, only channeled VL usage significantly reduces time to intubate compared to DL.

Number of Esophageal Intubations

**Figure 6:**
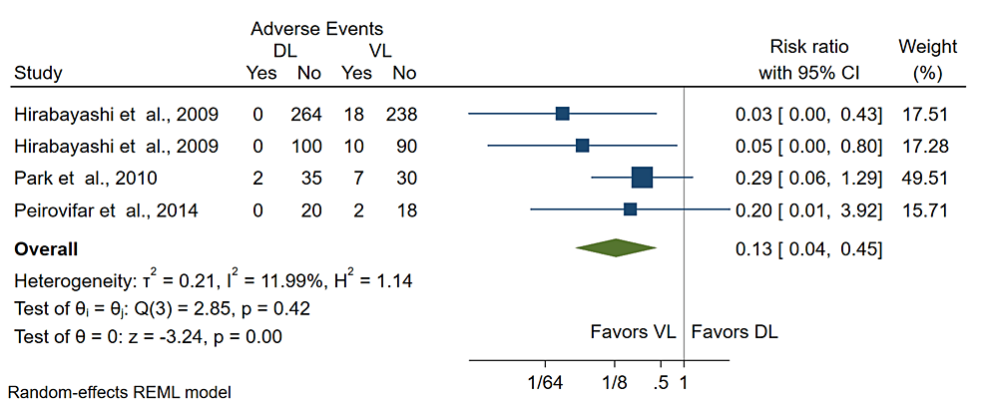
Forest plot of pooled risk ratios for the rate of esophageal intubation in novices using VL versus DL. CI: confidence interval; VL: video laryngoscopy; DL: direct laryngoscopy Hirabayashi et al. [16,17], Park et al. [21], Peirovifar et al. [22]

Four studies with 834 intubations (421 VL and 413 DL) reported data on the number of esophageal intubations. Two studies used Airtraq [17,21], one used Pentax [16], and one used Glidescope [22]. There was a significantly lower incidence of esophageal intubations with VL compared to DL (RR = 0.13; 95% CI = 0.04, 0.45) (Figure 6).

Discussion

This systematic review and meta-analysis suggests that novices have improved outcomes when using channeled VLs rather than DL. Additionally, VL is associated with improved success rates compared to DL when used by medical students.

VL allows one to visualize relevant anatomical structures without having to align the oral, pharyngeal, and laryngeal axes [25-27]. Whether this improved view translates to improved performance when intubating is affected by several factors, including the type of blade used [14,19]. However, laryngoscope blade type is often not considered in systematic reviews that compare VL and DL, and thus, we wanted to include this comparison [28]. Previous studies between VLs have shown improved outcomes with channeled devices, especially among inexperienced intubators [7,29]. Conversely, other studies have demonstrated that channeled and non-channeled blades are equally useful and suggest that device success is dependent on the overall structure, such as blade shape and camera position [30]. We found that non-channeled VLs do not improve outcomes over DL, and this may be due to difficulty in navigating the endotracheal tube despite the visualization of the glottis [14,19]. Channeled VL requires less complex maneuvering and decreases cognitive load, creating a safer learning environment [7,11].

Improved proficiency with VL compared to DL is notable in medical students. Medical students likely had the least amount of exposure to intubation out of all novice types and can be considered true novices. Their success with VL may be related to reduced cognitive stress, as VL decreases physical demand, perceived workload, and reaction times compared to DL [6]. This is consistent with other studies that have found that as intubators gain experience, they show less improvement with VL over DL compared to true novices [31]. Previous studies have also shown how early introduction of VL into the curriculum can improve outcomes in training [31,32]. Thus, VL use among novices in the early stages of training, such as medical students, is likely to be most beneficial for improving outcomes and patient safety.

Some studies have even shown that medical students learn faster when using VL rather than DL [33,34]. However, the limited amount of data on VL-acquired proficiency in novices over time prevents us from being able to provide a review of the learning curve of VL. Further RCTs are needed to determine how novices perform over a span of months or years with different types of VLs, as well as how training predominantly with these devices at first will affect other skills such as DL performance later on. Nonetheless, based on our current research, training medical students with VL allows for better outcomes and less concern for patient harm.

Our study also demonstrates a decreased rate of esophageal intubations with the use of VL over DL, which may be due to a superior view of the tube passing through the vocal cords. The small number of studies providing data for this outcome prevented sub-analyses of novice type or VL type.

Interestingly, we noticed that the study by Liu et al. had a higher DL first-pass success rate compared to the other studies [19]. This study also involved more clinical DL intubations in the training session and possibly skewed the results in favor of DL. However, a sensitivity analysis of the data without this study did not change our results enough to warrant its complete exclusion from this review. Additionally, the subjects in Liu et al. were referred to as “trainee anesthetists,” but the paper did not provide an explicit statement that they were novice trainees. Thus, we found it more prudent to evaluate them separately from the first-year residents. Had we included it in the resident subgroup, the results would be skewed in favor of DL among residents and in favor of VL among other novice clinicians.

Limitations

Our study is not without limitations. Time to intubate is a particularly heterogeneous variable given the differences in the definition of this outcome and the time cutoff that established failure. Although our results regarding time were generally consistent with the success rate, the variability of this outcome makes it challenging to draw meaningful conclusions. Differences in prior experience among novices are also a limitation; however, we attempted to address this via the sub-analysis. Some studies involved sequential intubation of patients by the same intubator [14-20,23]. While this increases the heterogeneity of the methods used in the studies, we determined that the small number of repeated intubations would not have a significant impact on the overall validity of this review in comparing VL and DL in novices, and we ensured that all intubators performed less than 20 intubations [3].

This review only presented a subset of VL types. With the constant development of new technologies, it is not possible to provide an all-encompassing review of available VL devices [35]. However, our review attempts to distinguish between two commonly used categories of VLs. Another limitation is the publication dates of the 10 studies, which range from 2009 to 2018. This may be due to a lack of continued research in this area as newer airway management tools are developed for specific use in emergent or critical care settings. Regardless of the publication date, the articles and devices in this review are important in the evaluation of VL, and studies surrounding these scopes are still being conducted even as advanced technologies are emerging [36].

This review only included intubation for elective surgeries in the operating room; application of these findings to practitioners performing difficult or emergent intubations is limited. Finally, due to the nature of performing intubation, we could not blind participants to the device being used, and all studies had a poor score regarding blinding in the bias assessment.

## Conclusions

In summary, the use of channeled VLs among novices results in improved outcomes compared to DL. This has benefits for patient safety since teaching with these devices will decrease the risk of adverse events. As experience level with clinical intubation increases, there appears to be less of a difference between the use of VL and DL, indicating that VL is especially useful in increasing patient safety when used by the medical student population, or true novices, as opposed to more advanced trainees. Further research is needed to quantify the learning curve for VL and determine how experience with these devices will impact overall intubation performance over time.

## Appendices

Search strategies for databases

PubMed

The search terms used were as follows: (“intubation” OR “endotracheal intubation” OR “orotracheal intubation” OR “endotracheal tube”) AND (“learning” OR “learning curve” OR “education” OR “educational measurement” OR “medical student” OR “student” OR “paramedical education” OR “paramedical personnel” OR “training” OR “novice” OR “resident” OR “residency education” OR “competence” OR “residency training” OR “nurse” OR “nurses” OR “anesthetist”) AND (“videorecording” OR “videolaryngoscopy” OR “videolaryngoscope” OR “video laryngoscopy” OR “videolaryngoscopy” OR “video” OR “laryngoscopy” OR “laryngoscope”).

Embase

The search terms used were as follows: (“intubation”/exp OR “intubation” OR “endotracheal intubation”/exp OR “endotracheal intubation” OR “endotracheal tube”/exp OR “endotracheal tube” OR “orotracheal intubation”/exp OR “orotracheal intubation”) AND (“learning”/exp OR “learning” OR “learning curve”/exp OR “learning curve” OR “education”/exp OR “education” OR “educational measurement”/exp OR “educational measurement” OR “medical student”/exp OR “medical student” OR “medical students”/exp OR “medical students” OR “paramedical education”/exp OR “paramedical education” OR “paramedical personnel”/exp OR “paramedical personnel” OR “novice” OR “resident”/exp OR “resident” OR “residency education”/exp OR “residency education” OR “competence”/exp OR “competence” OR “nurse”/exp OR “nurse” OR “nurses”/exp OR “nurses”) AND (“videorecording”/exp OR “videorecording” OR “endoscopic surgery”/exp OR “endoscopic surgery” OR “videolaryngoscopy”/exp OR “videolaryngoscopy” OR “videolaryngoscope”/exp OR “videolaryngoscope” OR “video”/exp OR “video” OR “laryngoscopy”/exp OR “laryngoscopy” OR “laryngoscope”/exp OR “laryngoscope”) AND (humans)/lim.

Cochrane Library

The search terms used were as follows: (video laryngoscopy in Title Abstract Keyword) AND (learning in All Text OR education in All Text OR competence in All Text OR resident in All Text OR medical student in All Text OR paramedic in All Text OR nurse in All Text) AND (intubation in All Text).

Web of Science

The search terms used were as follows: (intubation OR endotracheal OR intratracheal OR orotracheal) AND (learn OR experience OR inexperienced OR novice OR resident OR intern OR interns OR student OR skill OR teach OR taught OR train OR competency OR education OR instruction OR performance) AND (laryngoscope OR laryngoscopy OR videolaryngoscopy OR video laryngoscopy).
